# Development and application of a method to classify airborne pollen taxa concentration using light scattering data

**DOI:** 10.1038/s41598-021-01919-7

**Published:** 2021-11-16

**Authors:** Kenji Miki, Toshio Fujita, Norio Sahashi

**Affiliations:** 1 Faculty of Science and Technology, Keio University, 3-14-1, Hiyoshi, Kohoku-ku, Yokohama, Kanagawa 223-8522 Japan; 2 Tokyo Institute of Technology Earth-Life Science Institute, 2-12-1-IE-1, Ookayama, Meguro-ku, Tokyo, 152-8550 Japan; 3 Yamatronics Corporation, 2-1, Heiseicho, Yokosuka, Kanagawa 238-0013 Japan; 4 NPO Pollen Information Association, 2-7-4, Motookubo, Narashino, Chiba 275-0012 Japan

**Keywords:** Environmental sciences, Optics and photonics

## Abstract

Although automated pollen monitoring networks using laser optics are well-established in Japan, it is thought that these methods cannot distinguish between pollen counts when evaluating various pollen taxa. However, a method for distinguishing the pollen counts of two pollen taxa was recently developed. In this study, we applied such a method to field evaluate the data of the two main allergens in Japan, *Chamaecyparis obtusa* and *Cryptomeria japonica.* We showed that the method can distinguish between the pollen counts of these two species even when they are simultaneously present in the atmosphere. This result indicates that a method for automated and simple two pollen taxa monitoring with high spatial density can be developed using the existing pollen network.

## Introduction

Automated methods for evaluating airborne pollen concentrations have been developed by various research groups^1^. Examples include image recognition by deep learning^2–4^, neural networks^5^, fluorescence spectrometry^6^, microscopy image classification^7,8^, and nuclear magnetic resonance spectra^9^. Specifically, Plair Rapid-E is an automated pollen recognition device that uses scattering information and fluorescence spectra^10^. Swisens Poleno is an automated pollen monitoring system that uses pollen image data and a supervised deep learning method^11^. These systems may enable the nationwide pollen monitoring network to be automated, which is important for relaying airborne allergen distribution information at high spatial resolution^12–15^. Traditionally, pollen monitoring in the monitoring network was performed by counting pollen grains deposited on slides^16–18^, which is labour-intensive. In Japan, pollen counting is performed at more than 120 sites using laser optics, which are widely used and have improved the evaluation of airborne particle concentrations^19–24^. The automated pollen counter distributed in Japan is the KH-3000-01 (Yamatronics, Kanagawa, Japan) developed by Kawashima et al*.*^25,26^. The KH-3000-01 is widely used in Japan^27–30^ and has been occasionally tested some studies worldwide^31–33^. This system recognises pollen grains by irradiating the sampled particles with a laser beam and then evaluating the resulting light scattering intensity. Therefore, although the KH-3000-01 is robust, simple, and inexpensive, it may not distinguish the pollen count of different pollen taxa with similar light scattering characteristics, as it outputs the forward and side light scattering intensities. However, because various pollen taxa are simultaneously scattered in the atmosphere during some seasons^34^, it is important to monitor multiple pollen taxa. Although Matsuda and Kawashima^35^ suggested that pollen can be identified using the light scattering intensity when the data are analysed together with the spectrum of the pollen surface image, the method has not been applied to identify pollen grains. Miki et al*.*^36^ showed that pollen counts can be distinguished even for taxa with similar light scattering intensities by using the Automated Multi-taxa Pollen Counting Estimation System (AME system); however, because the experiment performed in the study was lab-based and the scattering data contained data from pollen grains, it remains unclear whether the AME system can be applied to actual field data, which are thought to be largely influenced by dust, meteorological factors, and set-up conditions. In this research, to investigate the applicability of the AME system, the field data of *Chamaecyparis obtusa* and *Cryptomeria japonica* were analysed using this system to establish an extensive and dense nationwide automated multi-taxon airborne pollen concentration monitoring network. We selected these taxa because they are the main springtime allergens in Japan and have been well-studied^37–41^.

## Methods

Light scattering data of *C. obtusa* and *C. japonica* were obtained using the AME system, which was first introduced in Miki et al*.*^36^. In the system, each pollen count was derived by solving the following equation:

1 $$\left(\frac{1}{{\int }_{0}^{4500}{P}_{\alpha }\left(x\right) {\mathrm{d}} x}\right){\int }_{a}^{b}{P}_{\alpha }\left(x\right) {\mathrm{d}} x {N}_{\alpha } + \left(\frac{1}{{\int }_{0}^{4500}{P}_{\beta }\left(x\right) {\mathrm{d}} x}\right){\int }_{a}^{b}{P}_{\beta }\left(x\right) {\mathrm{d}} x {N}_{\beta }={n}_{ab} \left(\frac{1}{{\int }_{0}^{4500}{P}_{\alpha }\left(x\right) {\mathrm{d}} x}\right){\int }_{c}^{d}{P}_{\alpha }\left(x\right) {\mathrm{d}} x {N}_{\alpha } + \left(\frac{1}{{\int }_{0}^{4500}{P}_{\beta }\left(x\right) {\mathrm{d}} x}\right){\int }_{c}^{d}{P}_{\beta }\left(x\right) {\mathrm{d}}x {N}_{\beta }={n}_{cd}$$

where $a$, $b$, $c$, $d$ are the light scattering intensities used as integration intervals, $P$ is the representative probability density as a function of the light scattering intensity of pollen taxa ($\alpha ,$ $\beta$), $N$ is the number of sampled pollen grains, $p$ is the number of the signals in the range of the integration intervals, and $n$ is the total number of sampled pollen grains in the integration interval.

Here, $P$ is the Gaussian function:

2 $$P\left(x\right)=\frac{1}{\sqrt{2\pi {\sigma }^{2}}} {\exp}\left\{-\frac{{\left(x-\mu \right)}^{2}}{2{\sigma }^{2}}\right\}.$$

The representative probability densities of *C. obtusa* and *C. japonica* were determined from 1500 light scattering data points for these species. The pollen was restored by Yamatronics, and the forward light scattering intensities of the two taxa were used for analysis. The integration intervals, ($a$-$b$) and ($c$-$d$), were set as the two points of voltage levels separated by 10 mV between 500 and 600 mV, and $c$ and $d$ were every set of two points of voltage levels separated by 10 mV between 600 and 700 mV, which are

$$ \left( {a,b} \right){:}\,\left\{ {\left( {{5}00,{ 51}0} \right), \, \left( {{5}00,{ 52}0} \right), \ldots \left( {{5}00,{ 6}00} \right), \, \left( {{51}0,{ 52}0} \right), \ldots \left( {{58}0,{ 6}00} \right), \, \left( {{59}0,{ 6}00} \right)} \right\} $$

$$ \left( {c,d} \right){:}\,\left\{ {\left( {{6}00,{ 61}0} \right), \, \left( {{6}00,{ 62}0} \right), \ldots \left( {{6}00,{ 7}00} \right), \, \left( {{61}0,{ 62}0} \right), \ldots \left( {{68}0,{ 7}00} \right), \, \left( {{69}0,{ 7}00} \right)} \right\}. $$

The number of each pollen grain was calculated based on every set of the integration intervals (*a*-*b* and *c*-*d*), and the calculation results were output if they were not negative. The number of pollen grains most frequently output as the calculation results were adopted as the number of pollen grains calculated by the AME system. Additionally, if the forward light scattering data of *C. obtusa* and *C. japonica* had the same variances and averages, obtaining meaningful outputs is not possible if the forward light scattering if the two species showed significant differences. Thus, the F test and *t* test. were used to test if the light scattering of the two species were significantly different.

### Evaluation test

**Figure 1:**
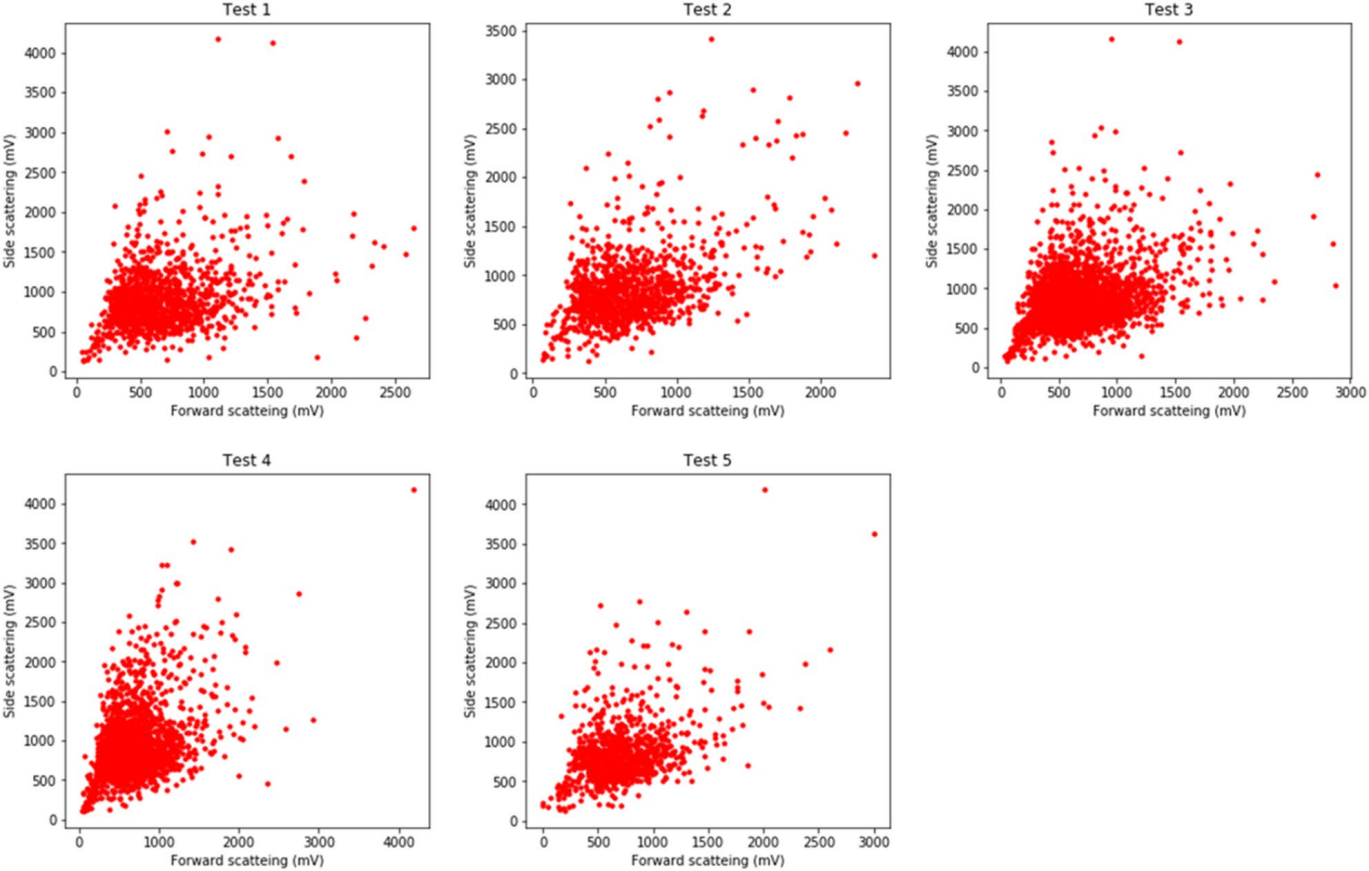
Test data in which the scattering intensities of *Chamaecyparis obtusa* and *Cryptomeria japonica* are mixed.

In addition to the light scattering data used to determine the representative light scattering probability, *C. obtusa* pollen and *C. japonica* pollen were injected into the KH-3000-01 following the guidelines of the Japanese Ministry of the Environment to obtain light scattering signals without contamination (Fig. 1). Five test data were obtained by randomly choosing light scattering data from each taxon.

### Application for actual field data

**Figure 2:**
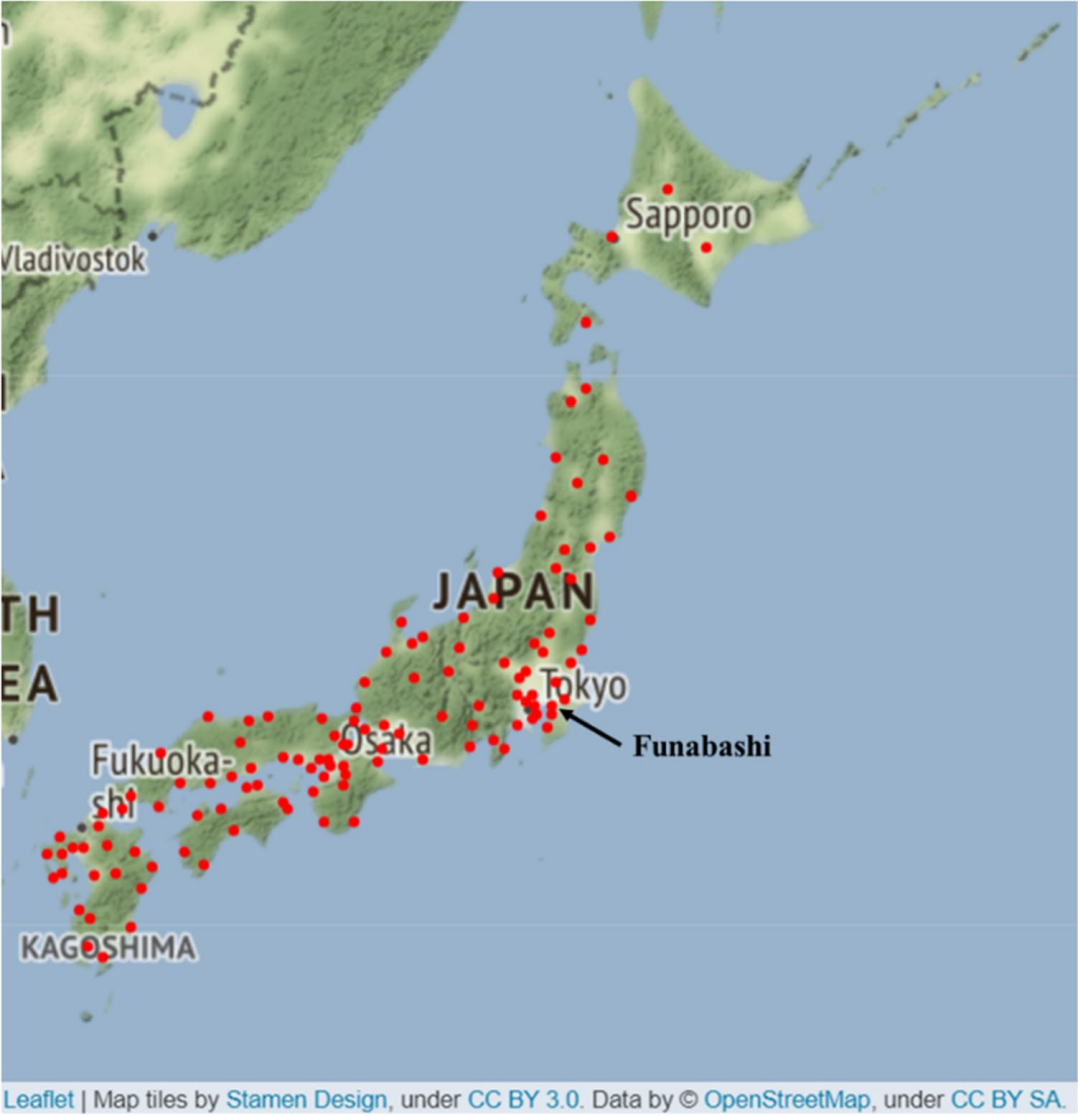
Installation locations of KH-3000-01 samplers in Japan (red dots) and experimental site at Funabashi. Generated by Stamen Maps http://maps.stamen.com/#watercolor/12/37.7706/-122.3782 (Accessed Oct. 22, 2021) and python-visualization, 2020. *Folium*, https://python-visualization.github.io/folium/ (Accessed Oct. 22, 2021). Installation locations were obtained from Japanese Ministry of the Environment  https://kafun.env.go.jp/  (in Japanese) (Accessed Oct. 22, 2021).

**Figure 3:**
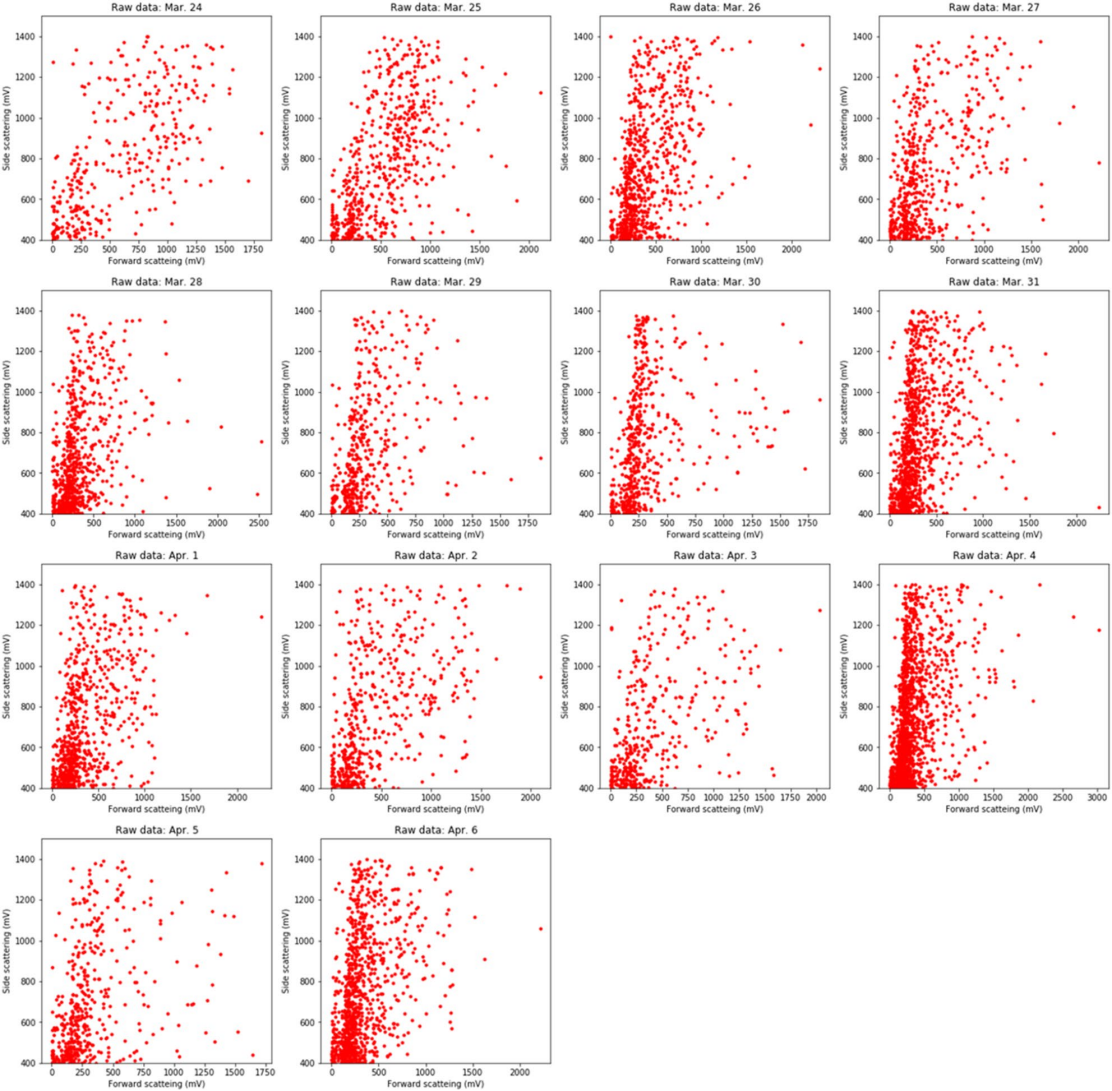
Field data from the KH-3000-01. Side-scattering data in the range of < 400 to > 1400 mV were excluded to avoid interference from dust data.

Pollen data were sampled according to the instructions of the Durham sampler and KH-3000-01 at Funabashi, where one of the pollen monitoring sites in Japan is located (Fig. 2), over 2 weeks from March 24 to April 6, 2012 (Fig. 3). Sampling by the Durham sampler was performed following the standardised protocol (PAAA and IAA protocol) of the NPO Pollen Information Association. The volumetric pollen concentrations evaluated by the KH-3000-01 and pollen deposition data evaluated by the Durham sampler were compared. Assuming that the pollen deposition speed of *C. obtusa* and *C. japonica* are constant, the relationship between pollen deposition and the airborne pollen concentration are constant. Thus, the correlation coefficients between the Durham sampler and AME system directly indicate the applicability of the AME system for pollen concentration estimation. Data from this sampling period were chosen because the period is the main season during which both *C. obtusa* and *C. japonica* are present. To avoid any impact of dust data on the light scattering data, data with side scattering intensities within 400–1400 mV were selected.

Because the KH-3000-01 samples air at a rate of 4.1 L min^−1^, the number of signals from the KH-3000-01 was converted to the airborne pollen concentration (m^−3^) using Eq. (3):

3 $${C}_{p}=\frac{1000\times {N}_{p}}{4.1\times 60\times 24}.$$

The confidence coefficient calculated using Eq. (4) is introduced as the criterion for determining the validity of the calculation results derived by the AME system.

4 $$Confident \; coefficient=\frac{L}{N}\times \frac{C}{L}\times P =\frac{CP}{N},$$

where $L$ is the number of outputs, $N$ is the total number of calculation results (3,025 in this experiment), $P$ is the number of outputs that show the estimated number of pollen grains, and $C$ is the total airborne pollen concentration calculated by the system which is the total of the estimated concentrations of *C. obtusa* and *C. japonica* each day. The confidence coefficient indicates the uniqueness of the outputs from the system. If each confidence coefficient of *C. obtusa* and *C. japonica* was below 2.0, the AME system’s calculation result was considered as invalid because the system was “not confident”.

## Results

**Figure 4:**
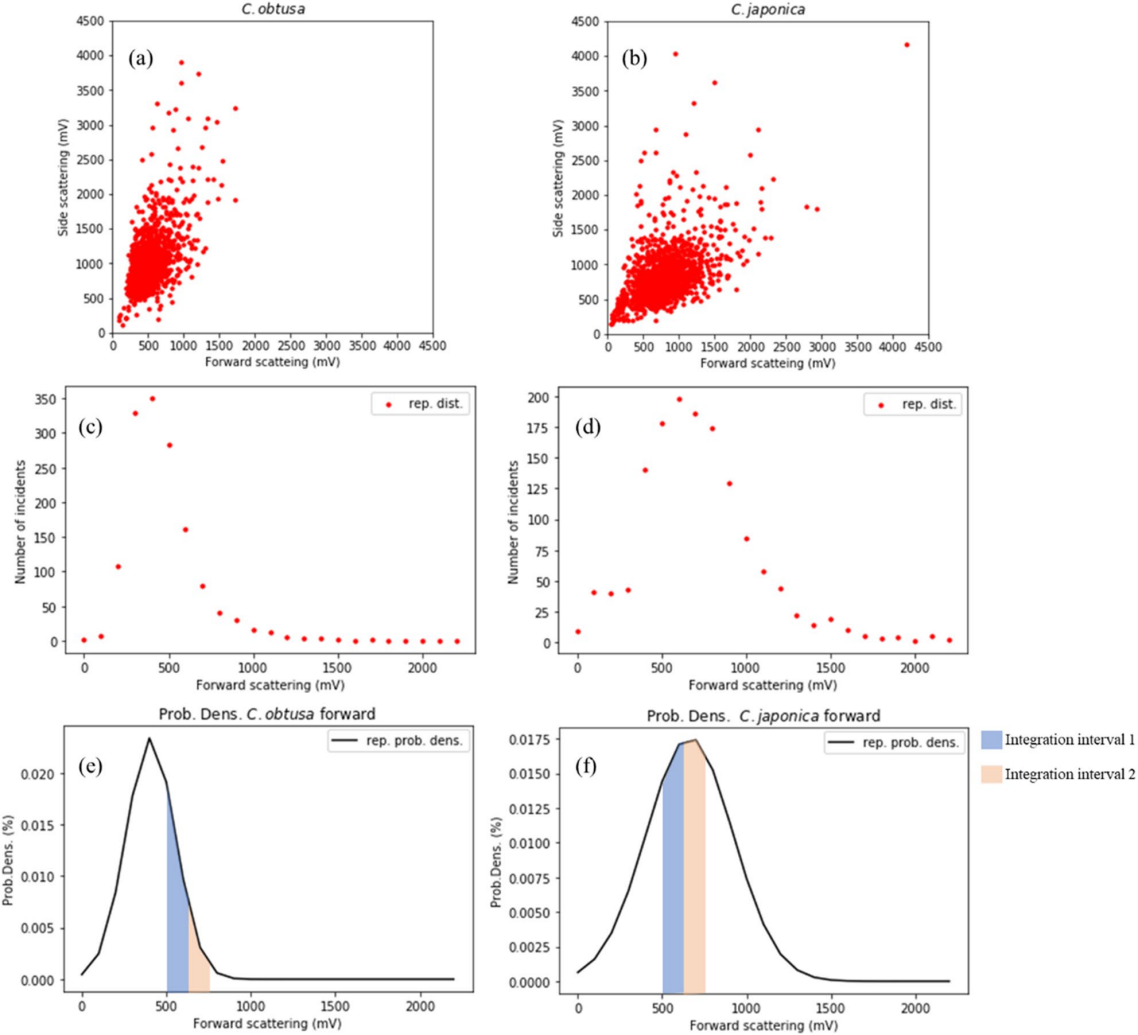
(**a**) Scattering intensity of *Chamaecyparis obtusa* and (**b**) *Cryptomeria japonica*, (**c**) number of forward-scattering signals of *C. obtusa* and (**d**) *C. japonica*, (**e**) probability density of forward-scattering signals of *C. obtusa* and (**f**) *C. japonica.*

We obtained 1500 points of raw light scattering data of *C. obtusa* and *C. japonica*. The representative light intensity data of *C. obtusa* and *C. japonica* were obtained by fitting the forward light scattering intensity distribution to the Gaussian function (Fig. 4).

The coefficients of the Gaussian functions for the light scattering of each taxon produced by curve fitting were derived as follows:

$${P}_{C. obtusa}{:}\,\left(\mu , \sigma \right)=\left(408, 145\right),$$

$${P}_{C. japonica}{:}\,\left( \mu , \sigma \right)=\left(662, 257\right).$$

Using the F test, the F boundary value, F value, and p value were derived as 1.09, 3.10, and below 0.05, respectively. In the *t* test, the t boundary value, t value, and p value were derived as 1.65, − 24.72, and below 0.05, respectively. Hence, the variance and average light scattering intensities of *C. obtusa* and *C. japonica* showed significant differences even though they overlapped.

### Evaluation test

**Figure 5:**
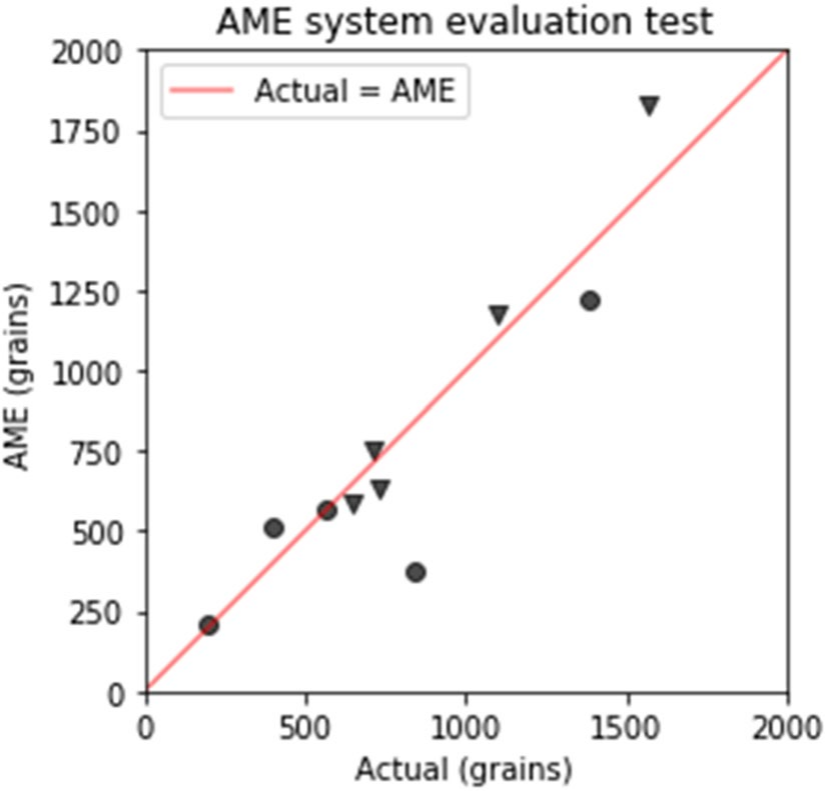
Results of five tests (circle: *Chamaecyparis obtusa*, triangle: *Cryptomeria japonica*. The red line represents the line on which the actual data and the estimation by the AME system are the same.

The evaluation test results showed that the AME system accurately calculated the number of pollen grains for each taxon (Fig. 5). Table 1 shows the actual and estimated numbers of *C. obtusa* and *C. japonica*. These results revealed that the AME system could distinguish the sampled number of pollen grains of *C. japonica* and *C. obtusa* with high accuracy when there was no dust contamination.

**Table 1:** Actual and estimated numbers of pollen grains.

	Actual_*C. obtusa*	Actual_*C. japonica*	AME_*C. obtusa*	AME_*C. japonica*
Test 1	562	647	570	590
Test 2	404	732	510	630
Test 3	845	1567	375	1825
Test 4	1383	1099	1225	1175
Test 5	200	715	210	750

### Application to field data

**Figure 6:**
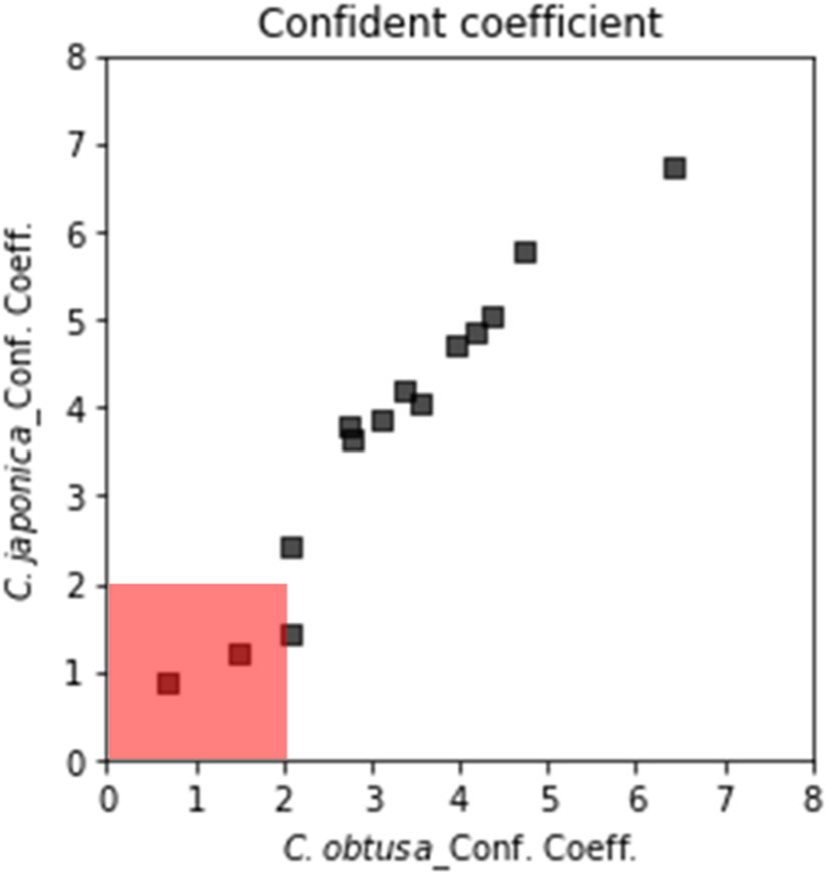
Results of confidence coefficients. If a confidence coefficient is in the red shaded area (> 2.0), the calculation result from the Automated Multi-taxa Pollen Counting Estimation System (AME) system was regarded as invalid.

**Figure 7:**
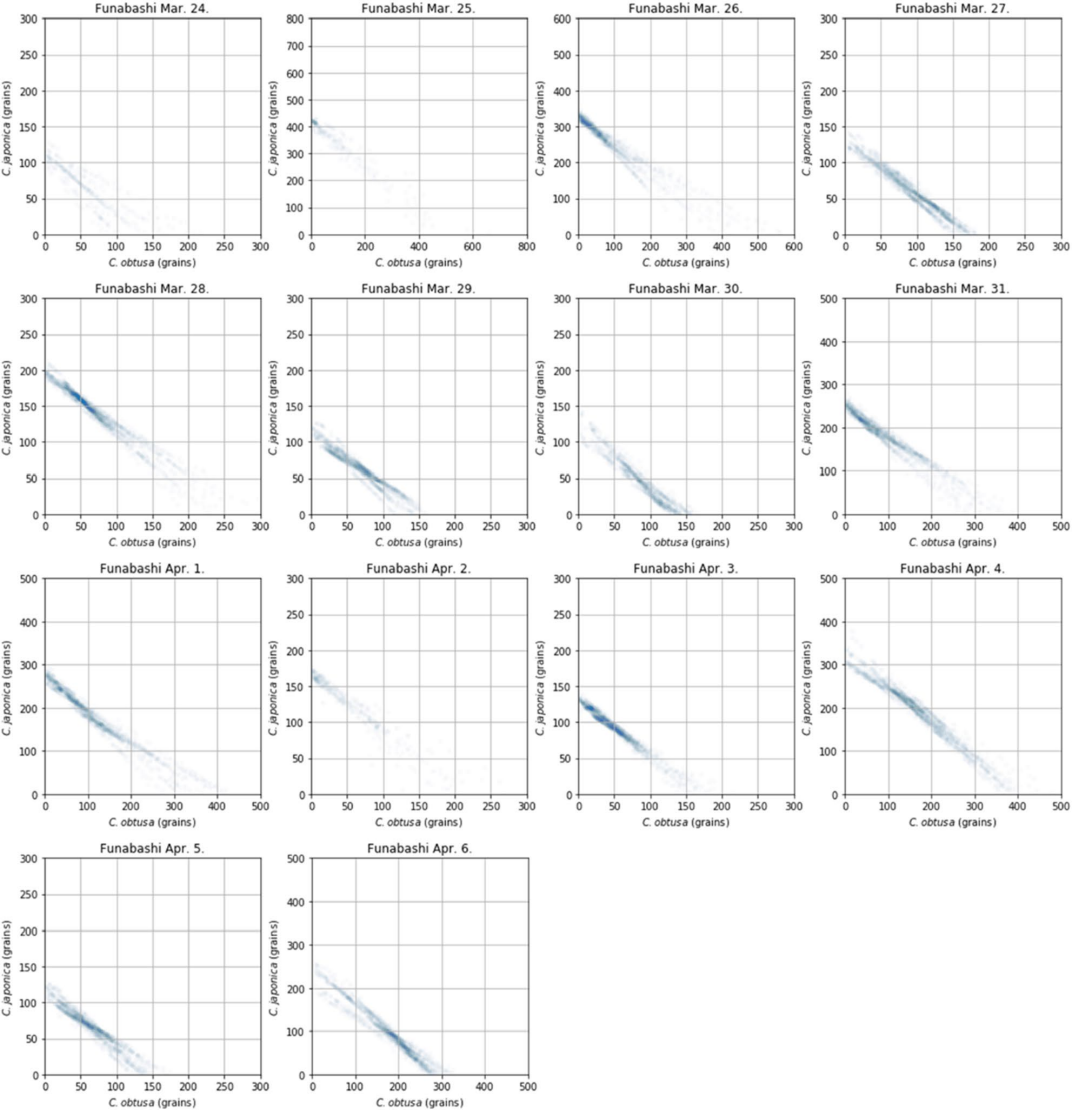
Outputs from the AME system (alpha = 0.01).

**Figure 8:**
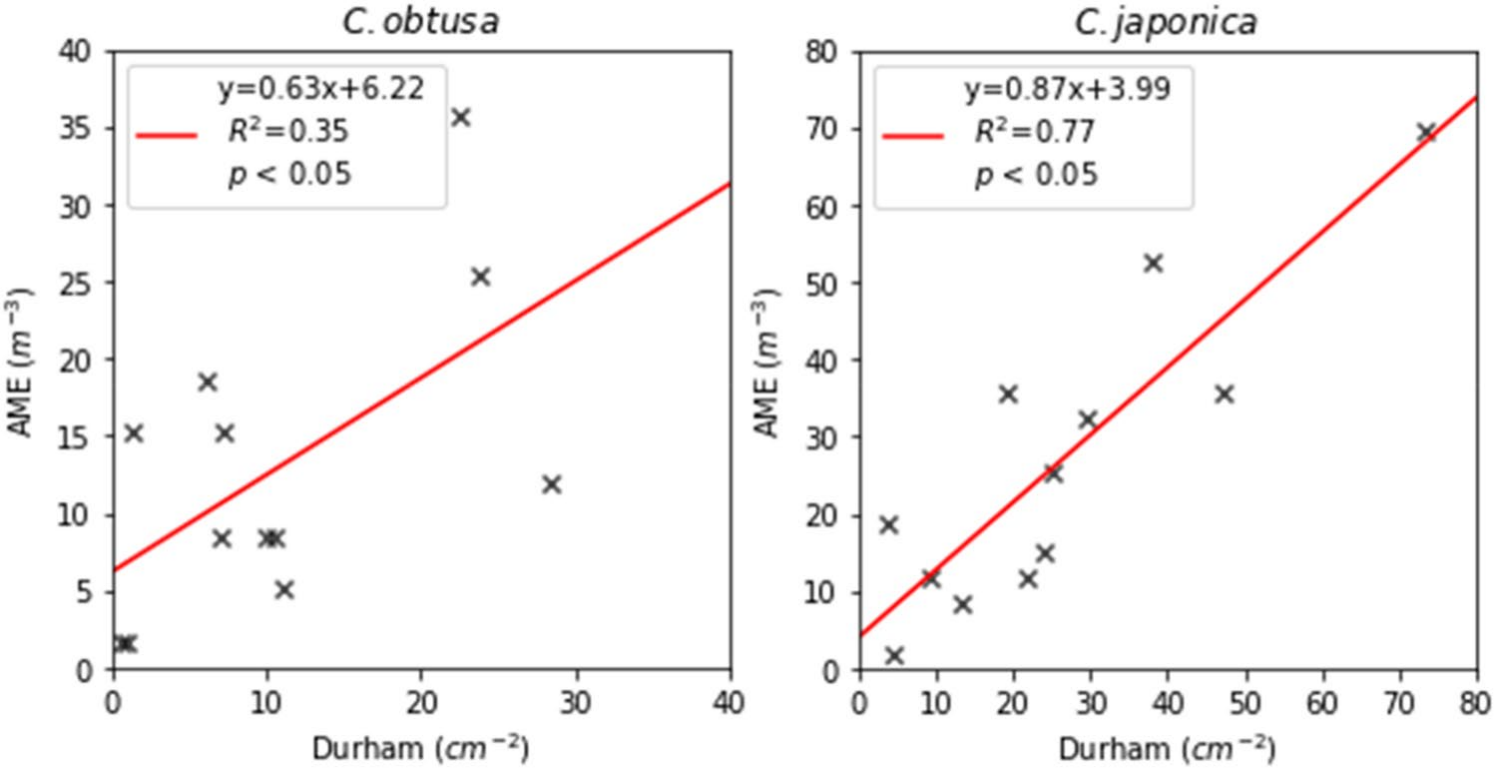
Relationship between estimation results of *Chamaecyparis obtusa* (left) and *Cryptomeria japonica* (right) by the Automated Multi-taxa Pollen Counting Estimation System (AME) system and Durham sampler.

According to the confidence coefficients derived from Eq. (4), the system was not confident about the results obtained on March 23 and April 2 (Table 2). When the confidence coefficient of one taxon was low, the other taxon tended to be low (Fig. 6). When the outputs were evaluated on a scatter plot with alpha = 0.01 (Fig. 7), a higher the confidence coefficient was found to correspond to a more unique number of pollen grains. Additionally, when the confident coefficient was below 2.0, the uniqueness of the output was low (March 24 and April 2). When the calculation results were compared with the number of pollen grains sampled by the Durham sampler, the determination coefficients between the Durham sampler and number of signals from KH-3000-01 was 0.20 (*C. obtusa*) and 0.45 (*C. japonica*). The determination coefficients between the Durham sampler and AME system were 0.35 (*C. obtusa*) and 0.77 (*C. japonica*) (Fig. 8). Thus, the correlation coefficients were significantly improved through the analysis.

**Table 2:** Summary of number of pollen grains and confidence coefficients.

Date	Durham (cm^−2^)		AME (m^−3^)		Confident coefficient		Confident	Valid plots
	*C. obtusa*	*C. japonica*	*C. obtusa*	*C. japonica*	*C. obtusa*	*C. japonica*		
24-Mar	1.2	52.2	8.5	15.2	0.67	0.89	No	435
25-Mar	0.6	73.5	1.7	69.4	2.07	1.43	Yes	309
26-Mar	0.9	38.0	1.7	52.5	4.37	5.03	Yes	1434
27-Mar	1.4	13.4	15.2	8.5	2.77	3.65	Yes	2081
28-Mar	7.0	25.1	8.5	25.4	6.44	6.72	Yes	2288
29-Mar	28.3	21.7	11.9	11.9	3.10	3.87	Yes	1844
30-Mar	6.2	4.6	18.6	1.7	2.07	2.44	Yes	1440
31-Mar	10.5	29.6	8.5	32.2	3.39	4.21	Yes	1877
1-Apr	7.3	47.1	15.2	35.6	3.56	4.03	Yes	1944
2-Apr	37.7	4.0	1.7	25.4	1.49	1.22	No	515
3-Apr	11.1	3.9	5.1	18.6	3.97	4.71	Yes	2019
4-Apr	23.8	19.3	25.4	35.6	4.74	5.79	Yes	2285
5-Apr	10.0	9.4	8.5	11.9	2.73	3.78	Yes	1907
6-Apr	22.4	24.1	35.6	15.2	4.17	4.85	Yes	1981

## Discussion

We found that the number of pollen grains of two pollen taxa can be distinguished using simple light scattering intensity data, even if the light scattering data overlaps. Although the integration intervals were fixed at one set of values (600, 800, 300, and 500 mV) by Miki et al*.*^36^, the output of the calculation fluctuated even when the integration intervals were fixed. Hence, adopting the most frequent output calculation results as the estimated number of pollen grains appears to be effective.

The test evaluation results showed that improving the device to better prevent dust from being sampled would improve the accuracy of the AME system. However, the AME system calculates the number of pollen grains based on the number of signals within the signal level of the integration interval. Hence, the number of airborne dust particles itself does not affect the accuracy of the AME system, but dust with optical characteristics similar to that of pollen grains should be excluded from the data to improve accuracy. The reason that the system worked better in calculating *C. japonica* than *C. obtusa* is thought to be that the KH-3000-01 equipment was originally designed only to detect *C. japonica*. Thus, updating the device can improve the analysis of *C. obtusa*. The sampling efficiencies of the KH-3000-01 and Durham samplers for *C. obtusa* and *C. japonica* are expected to differ from each other and from the correlation coefficient between the actual airborne pollen concentrations and pollen sampled on the Durham sampler. Hence, the AME system may not function better for *C. japonica* than for *C. obtusa*.

Although *C. japonica* and *C. obtusa* have approximately the same size (30 μm), the light scattering of the two species were significantly different. Previous studies indicated that the light scattering characteristic is strongly related to the morphology and surface spectrum of the pollen grains^35^,^42^. Thus, the AME system seems to be applicable to various pairs of pollen species with different sizes, shapes, and surface spectrums. This is also suggested in Miki et al.^36^ showing that the light scatterings of different pollen taxa are often significantly different.

Some studies focused on automated identification and counting using machine learning, which is expected to lead to highly accurate results^2–4^. However, as inexpensive and robust pollen samplers are currently being widely used, an automated system that can distinguish two different types of samples pollen grains taxa based on the simple light scattering intensity can be established as a nationwide automated multi-taxon pollen counting network by applying the system improved in this research to the existing network infrastructure. In addition, although the main two types of pollen in Japan were analysed, the AME system can be applied to the main taxa in other countries and regions by modifying the conditions of the system such as the integration intervals and light scattering probability density. Moreover, the system is applicable regardless of the pollen seasons.

## Conclusion

Field data on the airborne concentrations of two pollen taxa can be separately evaluated from simple light scattering data. This result indicates that a nationwide automated, inexpensive, and robust system with the potential to classify pollen concentrations from multiple taxa with high spatial density can be established.

## Data Availability

Airborne pollen concentration data are available at http://kafun.taiki.go.jp/. The pollen grains sampled by the Durham sampler are restored by NPO Pollen Information Association. Other data are available from the corresponding author on reasonable request.

## Code availability

The code used in this study is available from the corresponding author on reasonable request.
